# A Retrospective Analysis Investigating Whether Case Volume Experience of the Anesthesiologist Correlates with Intraoperative Efficiency for Joint Arthroplasty

**DOI:** 10.1007/s10916-023-02015-1

**Published:** 2023-11-16

**Authors:** Alvaro A. Macias, Dale N. Bongbong, Ruth S. Waterman, Sierra Simpson, Rodney A. Gabriel

**Affiliations:** 1 Division of Perioperative Informatics, Department of Anesthesiology, University of California San Diego, 9300 Campus Point Drive, MC7770, La Jolla, CA 92037-7770 USA; 2 School of Medicine, University of California San Diego, 9500 Gilman Dr, La Jolla, CA 92093 USA

**Keywords:** Joint arthroplasty, Intraoperative efficiency, Anesthesiologist volume, Spinal anesthesia

## Abstract

The objective of this retrospective study was to determine if there was an association between anesthesiology experience (e.g. historic case volume) and operating room (OR) efficiency times for lower extremity joint arthroplasty cases. The primary outcome was time from patient in the OR to anesthesia ready (i.e. after spinal or general anesthesia induction was complete). The secondary outcomes included time from anesthesia ready to surgical incision, and time from incision to closing completed. Mixed effects linear regression was performed, in which the random effect was the anesthesiology attending provider. There were 4,575 patients undergoing hip or knee arthroplasty included. There were 82 unique anesthesiology providers, in which the median [quartile] frequency of cases performed was 79 [45, 165]. On multivariable mixed effects linear regression – in which the primary independent variable (anesthesiologist case volume history for joint arthroplasty anesthesia) was log-transformed – the estimate for log-transformed case volume was − 0.91 (95% confidence interval [CI] -1.62, -0.20, P = 0.01). When modeling time from incision to closure complete, the estimate for log-transformed case volume was − 2.07 (95% -3.54, -0.06, P = 0.01). Thus, when comparing anesthesiologists with median case volume (79 cases) versus those with the lowest case volume (10 cases), the predicted difference in times added up to only approximately 6 min. If the purpose of faster anesthesia workflows was to open up more OR time to increase surgical volume in a given day, this study does not support the supposition that anesthesiologists with higher joint arthroplasty case volume would improve throughput.

## Introduction

With an aging global population [1], it is likely that demand for joint arthroplasty surgeries will increase over the next decade [2–4]. The surgical volume for total hip arthroplasty from 2020 to 2040 is projected to approach a three-fold increase from approximately 498,000 to 1,429,000 [5]. A potential problem with this projected rise is the possible limited availability of operating room (OR) time and personnel to keep up with this demand [6, 7].

There has been growing interest in optimizing OR efficiency and utilization to increase surgical volume. The implementation of workflows targeting such improvement can facilitate the availability of increased surgical volume on a given day. One potential solution is creating dedicated anesthesiology teams that can aid in optimizing OR times. Spinal anesthesia is a common anesthetic plan of choice for joint arthroplasties, and may be superior to general anesthesia in terms of hospital stay length, incidence of nausea [8], and overall complication rate [9]. Interestingly, controversial differences have been found in overall OR efficiency between spinal versus general anesthesia [10–12]. The use of separate block rooms to perform spinal anesthesia and the procedure itself have been identified to affect OR efficiency [13–16]. In a retrospective study analyzing > 40,000 patients undergoing joint arthroplasty, anesthesiologist volume and experience was not associated with postoperative complications or hospital length of stay [17]. However, there is a gap in the literature on the analysis of the association of a dedicated anesthesiology team on intraoperative efficiency.

More studies [10, 11] are needed that analyze the association of having a dedicated anesthesiology team to perform the spinal anesthetics and manage intraoperative care for arthroplasties and operating room efficiency. To study this question, we sought to determine if experience of the attending anesthesiologist with joint arthroplasty anesthesia (i.e. case volume in a given time period) would be associated with improved time metrics related to OR efficiency – specifically time from patient in OR to induction complete (e.g. after spinal or general anesthesia is completed). We hypothesized that anesthesiologist attendings with more experience with joint arthroplasty anesthesia (i.e. higher case volume) would have improved time OR efficiency metrics.

## Materials and Methods

### Study Sample

This retrospective study was approved by our institution’s Human Research Protections Program for the collection of data from our electronic medical record system and the informed consent requirement was waived. Data were collected retrospectively from the electronic medical record system from the University of California, San Diego Healthcare System. Data from all patients that underwent surgery from surgeons that performed hip and knee arthroplasty were extracted from 2018 to 2022. Cases that were not hip arthroplasty, knee arthroplasty, revision knee arthroplasty, or revision hip arthroplasty were removed. Cases with missing data for any of the time metrics or body mass index (BMI) were removed. Cases that were performed by an anesthesiology attending that had less than 10 total cases during the study period were also removed. The manuscript adhered to the applicable Enhancing the Quality of Transparency of Health Research guidelines for observational studies.

### Primary Objective and Data Collection

The primary objective of this study was to determine if there was an association between anesthesiologist case volume (total hip/knee arthroplasty cases performed during the study period) and the duration of time between patient arrival to the OR and to anesthesia ready.

Secondary outcomes included time from anesthesia ready to surgical incision and time from surgical incision to closure complete. The OR efficiency metrics included the following definitions, in accordance with the Association of Anesthesia Clinical Directors Procedural Times Glossary [18]:

1. Time to patient in the operating room to anesthesia ready – this time period represented when patient had entered the room, positioned for anesthesia induction, surgical briefing had occurred, and then spinal or general anesthesia induction completed.
2. Time from anesthesia ready to surgical incision – this time period represented positioning patient for surgery after induction, sterile preparation and draping, to the point when incision was about to start.
3. Time from incision to closing completed – this time period represented the entire duration of the surgery from surgical incision to when closing had been completed.

These time points were routinely documented from nursing records. The primary independent variable was anesthesiologists’ total case volume for hip/knee arthroplasty anesthetics during the defined study period. The anesthesiologist was defined as the supervising anesthesiology attending on record who started the case. The anesthesiology team structure, however, was either solo anesthesiology attending provider, certified registered nurse anesthetist (CRNA) supervision (in which 3 rooms are being supervised), or anesthesiology resident supervision (in which 2 rooms are being supervised). To control for potential confounders, other variables collected included surgical procedure (total hip arthroplasty with a posterolateral approach, total hip arthroplasty with an anterior approach, total knee arthroplasty, unicompartmental knee arthroplasty, revision total hip arthroplasty, and revision total knee arthroplasty), surgeon (labeled as surgeon A, B, and C), American Society of Anesthesiologists Physical Status (ASA PS) classification score, patient age (years), BMI, sex, number of years attending anesthesiologist had been in practice (years since completion of last trainee year), primary anesthesia type (spinal anesthesia, epidural anesthesia, general anesthesia, spinal conversion to general anesthesia), receipt of preoperative peripheral nerve block, and anesthesiology team structure (solo attending, CRNA supervision, resident supervision). Of note, at our institution, among hip and knee arthroplasty patients, peripheral nerve blocks are only performed for knee arthroplasty. Peripheral nerve blocks are performed in the preoperative hold area prior to patient entering the OR. All spinals were performed in the operating room and not in the preoperative holding area.

### Statistical Analysis

R Statistical Programming Language (version 4.2.2) was used for all statistical analyses. Initially, we modeled the fit of case volume to our primary outcome – time from patient arrival in OR to anesthesia ready – to determine how best to model the relationship in subsequent regression analyses. We compared the linear model, log-transformation of case volume, and quadratic transformation of case volume – the model with the highest *R*^*2*^ was chosen. Next, we performed linear mixed models fit by restricted maximum likelihood to model the association of case volume to each anesthesia control time outcome. The random effect was the anesthesiology attending on record. The primary fixed effect was case volume. The multivariable models controlled for the following fixed effects: surgical procedure, surgeon, ASA PS classification score, patient age, BMI, sex, number of years attending anesthesiologist had been in practice (since graduation of last trainee year), primary anesthesia type, receipt of preoperative peripheral nerve block, and anesthesiology team structure. The estimates, its 95% confidence intervals (CI) and P-values were reported for each independent variable. Because we were assessing three different time metrics (time from patient arrival to OR to anesthesia ready, time from anesthesia ready to incision, and time from incision to closure complete), we chose a P-value < 0.017 as statistically significant (i.e. 0.05 divided by 3). For the mixed effects models, we reported the intraclass correlation coefficient (ICC) and its 95% CI.

## Results

**Fig. 1:**
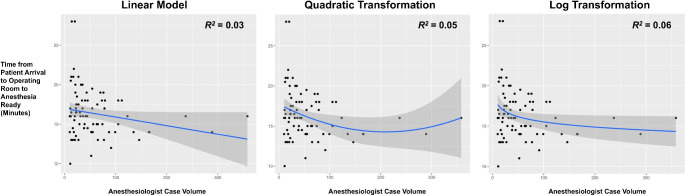
An assessment of the non-linear versus linear relationship of anesthesiologist case volume experience with joint arthroplasty anesthesia versus time from patient arrival to operating room to anesthesia ready. Fit between the non-transformed, log-transformed, and quadratic-transformed case volume variable with the anesthesia control time was assessed and the respective *R*^*2*^ was calculated for each

After exclusion, there were 4,575 joint arthroplasty surgeries included in the final study population (Table 1). There were 82 unique anesthesiology providers, in which the median [quartile] frequency of cases performed was 79 [45, 165], with a range of 10 to 358. Next, we evaluated the relationship between anesthesiologist case volume versus the time between patient in OR to anesthesia ready (Fig. 1). A log-transformation of case volume fit best (*R*^*2*^ = 0.06, P = 0.02) compared to linear model (*R*^*2*^ = 0.03, P = 0.06) and quadratic model (*R*^*2*^ = 0.05, P = 0.03). Thus, for the subsequent mixed effect regression models, case volume was log-transformed.

**Table 1:** Baseline characteristics of all patients in the study population

		n (%)
Total		4,575
Patient in OR to Anesthesia Ready (min), median [quartile]		15 [12, 22]
Anesthesia Ready to Incision (min), median [quartile]		23 [18, 29]
Incision to Close (min), median [quartile]		106 [91, 125]
Surgical Procedure		
	Total Hip Arthroplasty, Posterolateral Approach	1035 (22.6)
	Total Hip Arthroplasty, Anterior Approach	1281 (28.0)
	Total Knee Arthroplasty	1486 (32.5)
	Unicompartmental Knee Arthroplasty	200 (4.4)
	Revision Total Hip Arthroplasty	255 (5.6)
	Revision Total Knee Arthroplasty	318 (7.0)
Surgeon		
	A	2,063 (45.1)
	B	1177 (25.7)
	C	1335 (29.2)
Primary Anesthesia Type		
	Spinal Anesthesia	2,836 (62.0)
	Epidural Anesthesia	220 (4.8)
	General Anesthesia	1444 (31.6)
	Spinal Converted to General Anesthesia	75 (1.6)
Receipt of Preoperative Peripheral Nerve Block		1897 (41.5)
Anesthesiology Team Structure		
	Solo Attending	1762 (38.5)
	CRNA Supervision	1870 (40.9)
	Resident Supervision	943 (20.6)
# of Years Anesthesiology Attending in Practice, median [quartile]		10 [6, 12]
ASA PS Classification Score		
	Healthy (ASA 1)	78 1.7)
	Mild Systemic Disease (ASA 2)	2431 (53.1)
	Severe Systemic Disease (ASA 3)	2030 (44.4)
	Incapacitating Disease (ASA 4)	36 (0.79)
Patient Age (years)		66 [59, 73]
Body Mass Index (kg/m2)		28.3 [24.9, 32.6]
Male Sex		2053 (44.9)

ASA PS, American Society of Anesthesiologist Physical Status

CRNA, certified registered nurse anesthetist

OR, operating room

### Primary Outcome

Initially, a mixed effect linear regression model was performed, in which the dependent variable was time from patient in OR to anesthesia ready, the random effect was the anesthesiology attending, and the only fixed effect for the initial univariate analysis was the log-transformed anesthesiologist case volume. The estimate (95% CI) for log-transformed case volume was − 0.97 (standard error 0.37, P = 0.01). The ICC was 0.067 (95% CI 0.042, 0.092). Next, we performed a multivariable mixed effect linear regression, in which we controlled for various confounders by including the following fixed effects: age, sex, BMI, ASA PS classification score, primary procedure, surgeon, type of anesthesiology team (solo attending, resident anesthesiologist supervision, or CRNA supervision), primary anesthesia type, years attending anesthesiologist was in practice, and use of preoperative peripheral nerve block (Table 2).

**Table 2:** Results of multivariable mixed effects linear regression modeling anesthesiologist’s case volume history for joint arthroplasty anesthesia to time from patient arrive to the operating room to anesthesia ready. The random effect was the attending anesthesiologist for the case

Time (Patient in Room to Anesthesia Ready)					
Coefficients		Estimate	95% Confidence Interval		P-value
			lower	upper	
Case Volume (# of cases during study period)					
	Log-Transformed(Case Volume)	-0.91	-1.62	-0.20	0.01
Surgical Procedure					
	Total Hip Arthroplasty, Posterolateral Approach	Reference			
	Total Hip Arthroplasty, Anterior Approach	0.78	-0.16	1.72	0.11
	Total Knee Arthroplasty	0.94	-0.80	2.68	0.29
	Unicompartmental Knee Arthroplasty	0.47	-1.57	2.51	0.65
	Revision Total Hip Arthroplasty	1.31	0.08	2.54	0.04
	Revision Total Knee Arthroplasty	2.16	0.22	4.10	0.03
Surgeon					
	A	Reference			
	B	1.26	0.46	2.06	0.002
	C	0.32	-0.44	1.08	0.42
Primary Anesthesia Type					
	Spinal Anesthesia	Reference			
	Epidural Anesthesia	0.01	-1.15	1.17	0.97
	General Anesthesia	-0.02	-0.55	0.51	0.92
	Spinal Converted to General Anesthesia	0.68	-1.30	2.66	0.49
Receipt of Preoperative Peripheral Nerve Block		-1.17	-2.84	0.50	0.17
Anesthesiology Team Structure					
	Solo Attending	Reference			
	CRNA Supervision	0.27	-0.28	0.82	0.35
	Resident Supervision	0.41	-0.28	1.10	0.24
# of Years Anesthesiology Attending in Practice		0.07	-0.03	0.17	0.15
ASA PS Classification Score					
	Healthy (ASA 1)	Reference			
	Mild Systemic Disease (ASA 2)	1.71	-0.27	3.69	0.09
	Severe Systemic Disease (ASA 3)	2.19	0.19	4.19	0.03
	Incapacitating Disease (ASA 4)	6.54	3.11	9.97	0.0002
Patient Age (years)		0.06	0.04	0.08	< 0.0001
Body Mass Index (kg/m2)		0.12	0.08	0.16	< 0.0001
Male Sex		0.05	-0.46	0.56	0.82

ASA PS, American Society of Anesthesiologist Physical Status

CRNA, certified registered nurse anesthetist

The estimate (95% CI) for log-transformed case volume was − 0.91 (95% CI -1.62, -0.20, P = 0.01). The ICC was 0.062 (95% CI 0.038, 0.089). Therefore, if we compared this time metric between the anesthesiologist with the median case volume (79 cases) to lowest case volume (10 cases), the predicted difference based on this model would be 1.88 min in favor of the anesthesiologist with the higher case volume. Furthermore, if we compared this time metric between the anesthesiologist with the highest case volume (358 cases) to lowest case volume (10 cases), the predicted difference based on this model would be 3.25 min in favor of the anesthesiologist with the higher case volume.

### Secondary Outcomes

Next, a mixed effect linear regression model was similarly performed, in which the dependent variable was time from anesthesia ready to incision, the random effect was the anesthesiology attending, and the only fixed effect was the log-transformed anesthesiologist case volume. The estimate for log-transformed case volume was − 0.09 (95% CI -0.72, 0.54, P = 0.77). The ICC was 0.049 (95% CI 0.027, 0.073). A multivariable mixed effect linear regression controlling for same fixed effects as our primary analysis was then performed (Table 3). There was no association between case volume and time from anesthesia ready to incision – in which the estimate for the log-transformed case volume was 0.04 (95% CI -0.51, 0.59, P = 0.88). The ICC was 0.040 (95% CI 0.022, 0.060).

**Table 3:** Results of multivariable mixed effects linear regression modeling anesthesiologist’s case volume history for joint arthroplasty anesthesia to time from anesthesia ready to surgical incision. The random effect was the attending anesthesiologist for the case

Time (Anesthesia Ready to Incision)					
Coefficients		Estimate	95% Confidence Interval		P-value
			lower	upper	
Case Volume (# of cases during study period)					
	Log-Transformed(Case Volume)	0.04	-0.51	0.59	0.88
Surgical Procedure					
	Total Hip Arthroplasty, Posterolateral Approach	Reference			
	Total Hip Arthroplasty, Anterior Approach	5.05	4.21	5.89	< 0.0001
	Total Knee Arthroplasty	-1.96	-3.55	-0.37	0.02
	Unicompartmental Knee Arthroplasty	-3.71	-5.55	-1.87	< 0.0001
	Revision Total Hip Arthroplasty	6.08	4.96	7.20	< 0.0001
	Revision Total Knee Arthroplasty	-0.85	-2.63	0.93	0.35
Surgeon					
	A	Reference			
	B	-2.35	-3.06	-1.64	< 0.0001
	C	-0.12	-0.83	0.59	0.74
Primary Anesthesia Type					
	Spinal Anesthesia	Reference			
	Epidural Anesthesia	0.42	-0.64	1.48	0.44
	General Anesthesia	0.02	-0.47	0.51	0.93
	Spinal Converted to General Anesthesia	-0.28	-2.06	1.50	0.76
Receipt of Preoperative Peripheral Nerve Block		0.55	-0.96	2.06	0.48
Anesthesiology Team Structure					
	Solo Attending	Reference			
	CRNA Supervision	0.01	-0.50	0.52	0.96
	Resident Supervision	-0.16	-0.77	0.45	0.61
# of Years Anesthesiology Attending in Practice		0.17	0.09	0.25	< 0.0001
ASA PS Classification Score					
	Healthy (ASA 1)	Reference			
	Mild Systemic Disease (ASA 2)	-0.33	-2.11	1.45	0.72
	Severe Systemic Disease (ASA 3)	-0.32	-2.14	1.50	0.73
	Incapacitating Disease (ASA 4)	3.12	0.00	6.24	0.05
Patient Age (years)		-0.04	-0.06	-0.02	< 0.0001
Body Mass Index (kg/m2)		0.08	0.04	0.12	0.0005
Male Sex		0.09	-0.36	0.54	0.67

ASA PS, American Society of Anesthesiologist Physical Status

CRNA, certified registered nurse anesthetist

Next, a mixed effect linear regression model was similarly performed, in which the dependent variable was time from start of surgical incision to closure complete, the random effect was the anesthesiology attending, and the only fixed effect was the log-transformed anesthesiologist case volume. The estimate for log-transformed case volume was − 2.56 (95% CI -4.87, -0.25, P = 0.03). The ICC was 0.014 (95% CI 0.004, 0.022). A multivariable mixed effect linear regression controlling for the same fixed effects as our primary analysis was then performed (Table 4).

**Table 4:** Results of multivariable mixed effects linear regression modeling anesthesiologist’s case volume history for joint arthroplasty anesthesia to time from surgical incision to closure complete. The random effect was the attending anesthesiologist for the case

Time (Incision to Close)					
Coefficients		Estimate	95% Confidence Interval		P-value
			lower	upper	
Case Volume (# of cases during study period)					
	Log-Transformed(Case Volume)	-2.07	-3.54	-0.60	0.01
Surgical Procedure					
	Total Hip Arthroplasty, Posterolateral Approach	Reference			
	Total Hip Arthroplasty, Anterior Approach	10.02	6.04	14.00	< 0.0001
	Total Knee Arthroplasty	11.73	4.30	19.16	0.002
	Unicompartmental Knee Arthroplasty	-14.88	-23.52	-6.24	0.0007
	Revision Total Hip Arthroplasty	87.79	82.56	93.02	< 0.0001
	Revision Total Knee Arthroplasty	74.95	66.62	83.28	< 0.0001
Surgeon					
	A	Reference			
	B	-16.49	-19.70	-13.28	< 0.0001
	C	-1.39	-4.66	1.88	0.41
Primary Anesthesia Type					
	Spinal Anesthesia	Reference			
	Epidural Anesthesia	-0.15	-5.13	4.83	0.95
	General Anesthesia	0.42	-1.89	2.73	0.72
	Spinal Converted to General Anesthesia	-4.47	-12.80	3.86	0.29
Receipt of Preoperative Peripheral Nerve Block		-1.47	-8.55	5.61	0.69
Anesthesiology Team Structure					
	Solo Attending	Reference			
	CRNA Supervision	-0.96	-3.33	1.41	0.43
	Resident Supervision	-1.73	-4.61	1.15	0.24
# of Years Anesthesiology Attending in Practice		0.16	-0.06	0.38	0.15
ASA PS Classification Score					
	Healthy (ASA 1)	Reference			
	Mild Systemic Disease (ASA 2)	0.79	-7.52	9.10	0.85
	Severe Systemic Disease (ASA 3)	1.07	-7.42	9.56	0.81
	Incapacitating Disease (ASA 4)	11.56	-3.04	26.16	0.12
Patient Age (years)		-0.46	-0.56	-0.36	< 0.0001
Body Mass Index (kg/m2)		0.71	0.49	0.93	< 0.0001
Male Sex		5.24	3.10	7.38	< 0.0001

ASA PS, American Society of Anesthesiologist Physical Status

CRNA, certified registered nurse anesthetist

The estimate for log-transformed case volume was − 2.07 (95% CI -3.54, -0.60, P = 0.011). The ICC was 0.013 (95% CI 0.003, 0.020). Therefore, if we compared this time metric between the anesthesiologist with the median case volume (79 cases) to lowest case volume (10 cases), the predicted difference based on this model would be 4.28 min in favor of the anesthesiologist with the higher case volume. Furthermore, if we compared this time metric between the anesthesiologist with the highest case volume (358 cases) to lowest case volume (10 cases), the predicted difference based on this model would be 7.4 min in favor of the anesthesiologist with the higher case volume.

## Discussion

In this analysis, we found that higher case volume for joint arthroplasty anesthesia for the attending anesthesiologist was associated with decreased times in both time from patient arrival to OR and anesthesia ready and time from incision to closure complete. While the findings were statistically significant, the number of minutes saved likely does not transfer to any clinically significant changes. For example, when we compared anesthesiologists with median case volume (79 cases) versus those with the lowest case volume (10 cases), the predicted difference in times added up to only ~ 6 min. Furthermore, the mixed effect models had low calculated ICCs (< 0.1), which suggested little similarity within each cluster (i.e. anesthesiologist). The results suggest that there may be some influence of the individual anesthesiology provider and their historic case volume in relation to intraoperative efficiency for joint arthroplasty cases, but the influence is small. If the purpose of faster anesthesia workflows was to open up more OR time to increase surgical volume in a given day, this study does not support the supposition that anesthesiologists with higher joint arthroplasty case volume would improve throughput. Of note, our study did not measure perioperative complication rates or postoperative outcomes as it relates to an anesthesiologist’s case volume experience. Thus, while this study does not support need for higher volume anesthesiologists to improve OR throughput for joint arthroplasty anesthesia, more studies are needed to determine if there is an impact on improved patient outcomes.

Some authors have described the benefit of a dedicated group of regional anesthesiologists to perform spinal anesthetics to maximize efficiency and minimize complications [11]. In this study, the association of anesthesiologist case volume and time between patient OR arrival and anesthesia ready was small, thus this may not translate into any real-time savings when performing joint arthroplasty. In fact, ASA PS classification score had the largest impact on time on anesthesia induction – approximately 7 min added in patients that were ASA PS 4. Others have found that parallel processing was beneficial when a dedicated bay in the preoperative area was used to perform spinal anesthesia before a patient was brought into the OR [11, 16, 19]. For instance, Smith et al. found that nonoperative time decreased by 36 min (or 50%) and operative time decreased by 14 min (12%) for each case with this protocol [16]. While this is one solution, it would be dependent on the availability of extra anesthesia teams, nursing, and space resources.

Although the results of this study do not promote the need for anesthesiology teams consisting of those with the most experience in joint arthroplasty anesthetics, it is still vital to prioritize technical competency in providing anesthetic care for these patients. There was also controversy if spinal anesthesia may improve outcomes for joint arthroplasty patients by reducing morbidity and mortality [20]. The REGAIN trial showed that spinal anesthesia did not increase survival or recovery of ambulation at 60 days after surgical repair for hip fractures when compared to general anesthesia [21]. In contrast, Memtsoudis showed that the use of neuraxial anesthesia for primary joint arthroplasty might confer a protective effect, while decreasing the incidence of thromboembolism, postoperative systemic infection [22], surgical time, and blood loss [23, 24].

While the clinical implication of spinal anesthesia is becoming more apparent, the implication on efficiency and money-saving initiatives are unclear. The average OR cost is around $36–37 per minute [25]. The operative time (block time) is fixed for a particular surgeon or service. When looking at OR efficiency, spinal anesthesia has been shown to increase the time to prepare the patient for surgery and total preoperative time while the time to remove the patient from the OR was significantly reduced. However, these differences cancel out when considering total OR time compared to general anesthesia [12].

Spinal anesthesia can be more challenging to perform due to the inherent characteristics of every patient and, as a result, could be less predictable in onset; however, once a surgical block is established, then spinal anesthesia may overall be less involved (compared to general anesthesia), and time related to emergence is minimal in the OR when compared to general anesthesia. Park showed that spinal anesthesia was associated with mild overall time saving compared to general anesthesia [26] and Chandler found no overall differences in nonoperative time between spinal anesthesia and general anesthesia [12].

This study focused on measuring the association of joint arthroplasty case volume experience of anesthesiologists with intraoperative efficiency. No meaningful clinical differences were found, as noted before. Actual time-saving initiatives may come from other approaches to OR times. Having a dedicated bay to perform regional and neuraxial anesthesia in the preoperative area has consistently shown adequate time and money savings [18, 25]. Furthermore, reducing surgical skin time could potentially have the largest impact on OR time saving and thus the potential to increase OR volume. Furthermore, trainee and CRNA involvement was measured, and we found no differences in intraoperative efficiency. Previous literature has reported trainee involvement to have no effect on anesthesia-controlled times [27] and minimal effect on emergence time [28].

We recognize that the study has some limitations. Given the retrospective nature of the study, there are inherent limitations related to accuracy of data. For example, OR time metrics were recorded by the circulating nurse, which could potentially introduce bias. However, this was done systematically and uniformly and potentially unlikely to skew the results to certain anesthesiology providers. To further validate the results of this study, a prospectively-designed clinical trial could better demonstrate the impact of having specialty anesthesiology teams for this surgical population, and whether this could improve overall OR utilization. More studies are needed to identify if OR and anesthesia-controlled times are linked to a particular anesthesia technique or more related to the resources and space available to implement more parallel processing. Another limitation is that we did not study association of case volume experience with postoperative outcomes, such as complications or hospital length of stay. These are important metrics that may be affected by the anesthesia care. Thus, our study is limited to providing data regarding the association of case volume experience with OR efficiency, but not postoperative outcomes.

In conclusion, based on this retrospective study of nearly 5,000 surgical cases, there were statistically significant but not clinically significant associations between an anesthesiologists’ joint arthroplasty anesthesia volume and operating room time – specifically time between patient arrival to OR and anesthesia ready and time between start of surgical incision to closure complete. The differences were not clinically significance as the measured time saved would not be sufficient to effectively add more joint arthroplasty surgeries in a given OR day. This study would then suggest that dedicated anesthesiologists with the most joint arthroplasty anesthesia volume for a joint arthroplasty surgery may provide limited benefit for improving throughput. However, future studies should measure associations with perioperative complications and postoperative outcomes as it relates to the anesthesiologist’s case volume experience.
